# Pseudo-Interface Switching of a Two-Terminal TaO*_x_*/HfO_2_ Synaptic Device for Neuromorphic Applications

**DOI:** 10.3390/nano10081550

**Published:** 2020-08-07

**Authors:** Hojeong Ryu, Sungjun Kim

**Affiliations:** Division of Electronics and Electrical Engineering, Dongguk University, Seoul 04620, Korea; hojeong.ryu@dongguk.edu

**Keywords:** memristor, synapse device, neuromorphic computing, tantalum oxide, hafnium oxide

## Abstract

Memristor-type synaptic devices that can effectively emulate synaptic plasticity open up new directions for neuromorphic hardware systems. Here, a double high-k oxide structured memristor device (TaO*_x_*/HfO_2_) was fabricated, and its synaptic applications were characterized. Device deposition was confirmed through TEM imaging and EDS analysis. During the forming and set processes, switching of the memristor device can be divided into three types by compliance current and cycling control. Filamentary switching has strengths in terms of endurance and retention, but conductance is low. On the other hand, for interface-type switching, conductance is increased, but at the cost of endurance and retention. In order to overcome this dilemma, we proposed pseudo interface-type switching, and obtained excellent retention, decent endurance, and a variety of conductance levels that can be modulated by pulse response. The recognition rate calculated by the neural network simulation using the Fashion Modified National Institute of Standards and Technology database (MNIST) dataset, and the measured conductance values show that pseudo interface-type switching produces results that are similar to those of an interface-type device.

## 1. Introduction

Compared with von Neumann architecture, which has limitations, such as a bottleneck between processor and memory, the human brain can learn and process a complex task with low power consumption and parallel computation [1,2]. In recent years, with the advent of big data and artificial intelligence (AI), a greater amount of data that is more complex can be efficiently processed with lower amounts of energy [3,4,5,6,7]. In response to the demand for new computing architecture, a neuromorphic system has been proposed and self-learning neuromorphic chips have already been developed. Neurons and synapses are the key components necessary for learning and memory function in the human brain [8]. For computing, neurons integrate signals from adjacent neurons and generate output signals. Synapses are responsible for controlling the strength of connections between neurons. In the memristor, which is a two-terminal memory device, the value of conductance can be updated by varying the applied pulse signal, which has a role similar to that of a synapse in the nervous system. The memristor should be well-characterized to implement neuromorphic computing [9].

One representative memristor, oxide-based resistive switching memory, has been studied in depth due to its excellent performance in terms of endurance, retention, reliability, reproducibility, and compatibility with complementary metal oxide semiconductors (CMOSs) [10,11,12,13]. To date, reversible resistive properties have been reported using a large number of oxides. Among them, HfO_2_ and Ta_2_O_5_ are the leading oxide materials that show outstanding memory characteristics [14,15,16]. In particular, when the oxide layer is deposited in two or three multi-layers, memory characteristics are improved because the layers act as oxygen reservoirs or tunnel barriers. For example, when HfO_2_ and Ta_2_O_5_ are stacked, HfO_2_ can be used as a switching layer and Ta_2_O_5_ can be used as an oxygen exchange layer [17,18]. In addition, as one dielectric is not fully percolated even in the low-resistance state (LRS) state, an asymmetric current-voltage (I-V) curve can be obtained by the difference in work function between the electrodes. This is advantageous because the current at one side has a rectifying action, but the reproducible nonvolatile memory properties are not observed at too low of a current level.

In this work, we fabricated a two-terminal Pt/TaO*_x_*/HfO_2_/TiN memristor device. The TaO*_x_* layer, acting as oxygen reservoir, is responsible for interface-type switching, while the formation and rupture of conducting filaments occurs within the HfO_2_ layer [18]. Three types of switching (filamentary, interface-type, and pseudo-interface-type) were possible depending on the compliance current (CC) and cycling effect. Using three types of comparison studies, we identified differences in endurance, retention, and variability. For implementation of a neuromorphic system, we examined the pseudo-interface-type switching mode of Pt/TaO*_x_*/HfO_2_/TiN as a synaptic device, and a large number of multilevel values, as well as excellent retention characteristics, were demonstrated. Furthermore, we confirmed the feasibility of the neuromorphic hardware system when using pseudo-interface switching of Pt/TaO*_x_*/HfO_2_/TiN as a synaptic device, which is verified by pattern recognition accuracy in a neural network simulation.

## 2. Materials and Methods

100-nm-thick TiN was deposited on a SiO_2_/Si substrate as the bottom electrode. HfO_2_ as a high-K thin film material was deposited using atomic layer deposition (ALD). TEMAHf and H_2_O were used to form HfO_2_ thin films. One cycle of the HfO_2_ thin film formation process consisted of TEMAHf 0.5 s, purge 35 s, H_2_O 0.3 s, and purge 35 s. The number of depositions was set to 105 cycles for a thickness of 7 nm. The chamber temperature was set to 280 °C and the source temperature to 90 °C. Next, TaO_x_ was deposited using DC reactive sputtering, in which Ar and O_2_ gases were used to form the TaO_x_ thin film. The flow rates were Ar = 8 sccm and O_2_ = 12 sccm. The base pressure was 1.6 × 10^−6^ Torr and the deposition pressure was 1 mTorr. The DC power used was 500 W (pulsed DC, 50 kHz). Lastly, Pt, an inert metal, with the diameter of 100 μm was deposited as the top electrode by electron beam evaporation using a shadow mask. The electrical properties in the DC sweep and transient modes were measured using a semiconductor parameter analyzer (Keithley 4200-SCS and 4225-PMU ultrafast module, Solon, OH, USA). Transmission electron microscope (TEM) and energy-dispersive X-ray spectroscopy (EDS) was conducted by JEM-2100F, Tokyo, JAPAN).

During the measurements, the DC bias voltage and pulse were applied to the Pt top electrode and the TiN bottom electrode was grounded.

## 3. Results and Discussion

**Figure 1:**
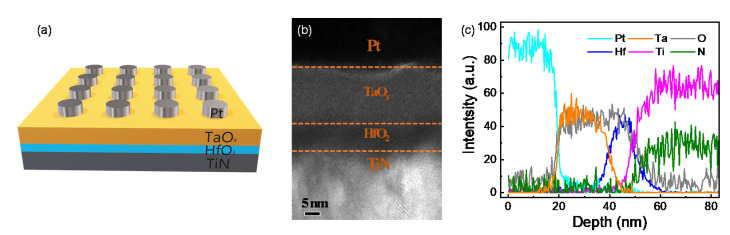
Device configuration of Pt/TaO*_x_*/HfO_2_/TiN capacitor structure: (**a**) Schematic; (**b**) TEM image; (**c**) EDS line scan.

Figure 1a shows the device structure of Pt/TaO*_x_*/HfO_2_/TiN memristor device. The thickness of TaO*_x_* and HfO_2_ is 8 nm and 18 nm that is confirmed by TEM. In addition, six elements (Pt, Ta, O, Hf, Ti, and N) per depth are well identified by EDS line scan.

**Figure 2:**
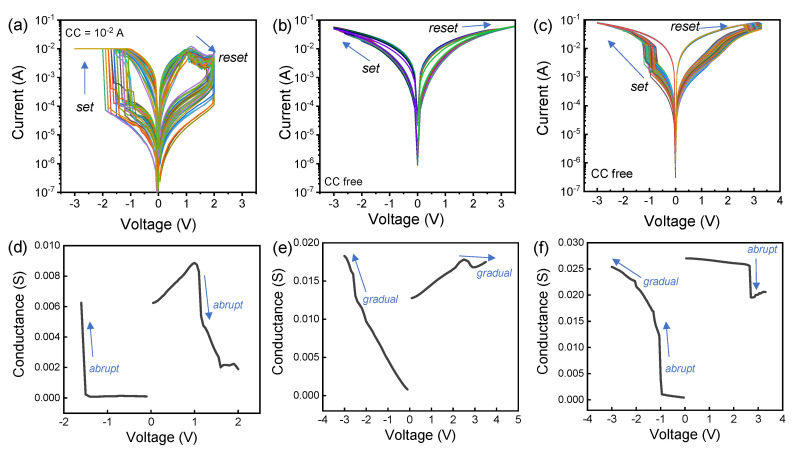
I-V characteristics of the TaO*_x_*/HfO_2_ memristor: (**a**) Non-volatile filamentary switching with compliance current (CC) = 10 mA; (**b**) non-volatile interface switching without CC; (**c**) non-volatile pseudo-interface switching without CC; Conductance-voltage (G-V) characteristics of the TaO*_x_*/HfO_2_ memristor. (**d**) non-volatile filamentary switching with CC = 10 mA; (**e**) non-volatile interface switching without CC; (**f**) non-volatile pseudo-interface switching without CC.

Figure 2 shows 3 different switching behaviors of the TaO*_x_*/HfO_2_ memristor. Figure 2a shows non-volatile filamentary switching with a CC of 10 mA. I-V curves at the lower CC values show self-rectification characteristics that were reported by similar RRAM stacks (Figure S1) [17,18]. However, at low current levels, it was difficult to obtain repetitive and uniform cell characteristics. A low-resistance state (LRS) is maintained after the abrupt set process and a high-resistance state (HRS) is maintained during the gradual reset process. Due to the non-uniform reset process, a large variation in HRS values is observed. Figure 2b shows interface-type I-V switching without CC in which the conductance or resistance value changes without a distinct current jump during the voltage sweep [19,20]. The on/off ratio can be slightly increased by increasing the voltage sweep range. However, there is a risk of device destruction due to negative-set phenomenon [21]. Figure 2c shows pseudo-interface-like switching in which slow conductance change is observed after an abrupt current jump. A higher on/off window is achieved with pseudo-interface switching compared to interface switching. The nature of the switching change is more clearly detectable through the linear conductance-voltage curve in Figure 2d–f. Note that pseudo-interface-like switching includes a gradual set process. The three switching modes are controllable by the CC during the forming and set processes (Figure S1) [22].

**Figure 3:**
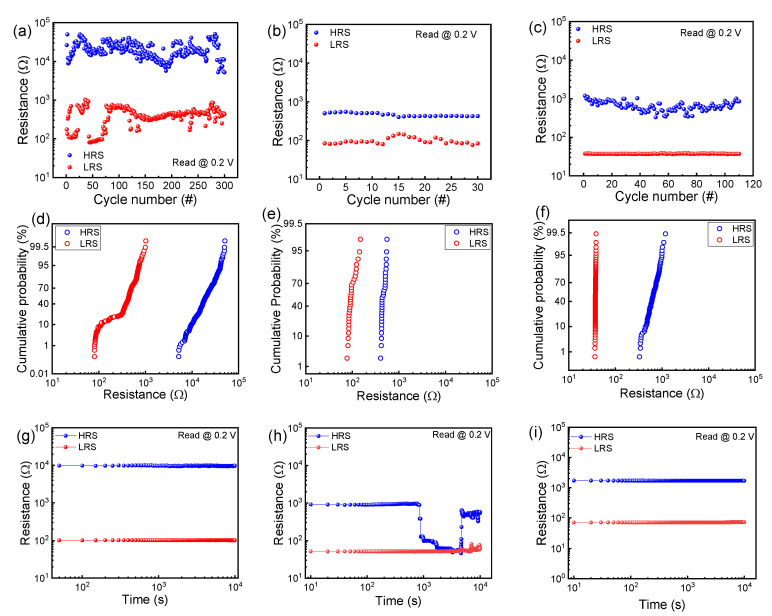
Reliability and variability characteristics of the TaO*_x_*/HfO_2_ memristor: Endurance test for (**a**) filamentary; (**b**) interface-type; and (**c**) pseudo-interface-type switching. Cumulative probability for (**d**) filamentary; (**e**) interface-type; and (**f**) pseudo-interface-type switching. Retention test for (**g**) filamentary; (**h**) interface-type; and (**i**) pseudo-interface-type switching.

Next, endurance, cumulative probability, and retention are investigated for the 3 different switching modes in the TaO*_x_*/HfO_2_ memristor to evaluate its reliability and variability [23]. Figure 3a–c show endurance tests for filamentary, interface-type, and pseudo interface-type switching, respectively. Figure 3d–f show the cumulative probability of HRS and LRS in filamentary, interface-type, and pseudo interface-type switching, respectively. The cycling number is highest but the variations in HRS and LRS are large in filamentary switching. Generation and rupture of the conducting filaments is a random process within the insulators, so the variation cannot be avoided. The current overshoot leads to variation in LRS during the set process and the HRS value is affected by the random filament rupture process in a manner that depends on the LRS resistance during the reset process. In the case of interface switching, the endurance cycling number is lowest even though HRS and LRS are the most uniform in Figure 3b,e. Unlike filamentary switching, where conductance is significantly altered due to the rapid generation of filaments, the conductance change of interface-type switching occurs as conducting defects are slowly controlled by the increase or decrease of the applied voltage. Pseudo-interface-type switching has an intermediate nature between those of the two preceding switching cases in terms of endurance, variability in HRS and LRS, and memory window (Figure 3c,f). Figure 3g–i show the retention properties for filamentary, interface-type, and pseudo-interface-type switching, respectively. Filamentary switching shows good retention characteristics; the values of LRS and HRS remain almost unchanged for 10,000 s (Figure 3g). On the other hand, in interface-type switching, the HRS resistance value suddenly decreases around 1000 s (threshold time), gradually decreases after that, and then shows large fluctuations. Repeated testing demonstrated collapse of the HRS value (Figure S2). Fluctuation occurs randomly, but the threshold time when the resistance changes rapidly is almost the same as 1000 s. Note that pseudo-interface-type switching shows good retention, like filamentary switching.

**Figure 4:**
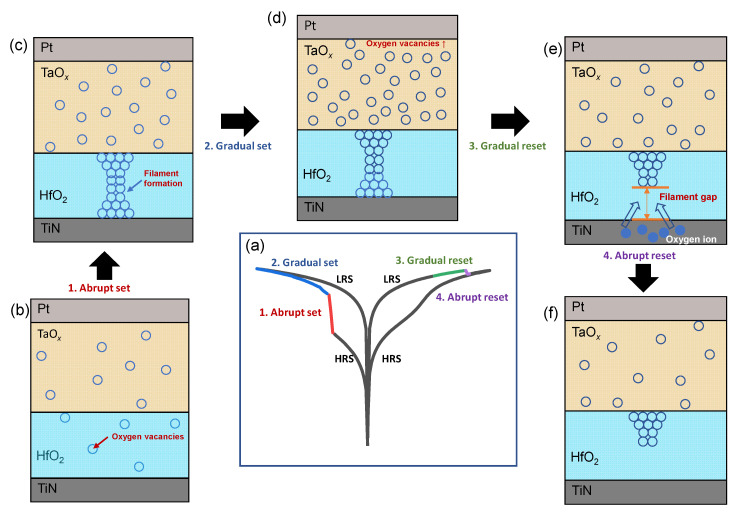
Switching model of the TaO*_x_*/HfO_2_ memristor using conducting defects and filaments: (**a**) I-V characteristics according to the voltage sweep; (**b**) high-resistance state (HRS); (**c**) low-resistance state (LRS) after abrupt set; (**d**) increased oxygen vacancies after an additional gradual set process; (**e**) reduced oxygen vacancies in the gradual reset process; (**f**) filament gap is open after an abrupt reset process.

Next, the possible switching mechanism of pseudo-interface-type switching is discussed (Figure 4). We match the I-V curve according to voltage and polarity to the switching model by referring to the existing models for HfO_2_ and TaO*_x_* in Figure 4a [24,25,26,27,28,29]. When a negative bias is applied to the top electrode in the initial state, it is divided into the HfO_2_ and TaO*_x_* layers in Figure 4b. Since the dielectric constant of HfO_2_ is smaller, a stronger field is applied to this layer and HfO_2_ is generally classified as a filamentary switching model (Figure 4c). The current shows self-compliance after an abrupt jump and the current gradually increases, suggesting that the number of oxygen vacancies mainly increase in TaO*_x_,* as shown in Figure 4d. Since the conducting filament is formed in the HfO_2_ layer, a higher voltage is applied to TaO*_x_*, which has a higher resistance, which produces additional oxygen vacancies. When a positive voltage of the opposite polarity is applied, oxygen ions move in the opposite direction, which causes oxygen vacancies to change in TaO*_x_* as the oxygen ions move toward the top electrode, as shown in Figure 4e. The movement of oxygen ions eventually leads to rupture of the filament inside HfO_2_, and, finally, abrupt reset is completed, as shown in Figure 4f.

**Figure 5:**
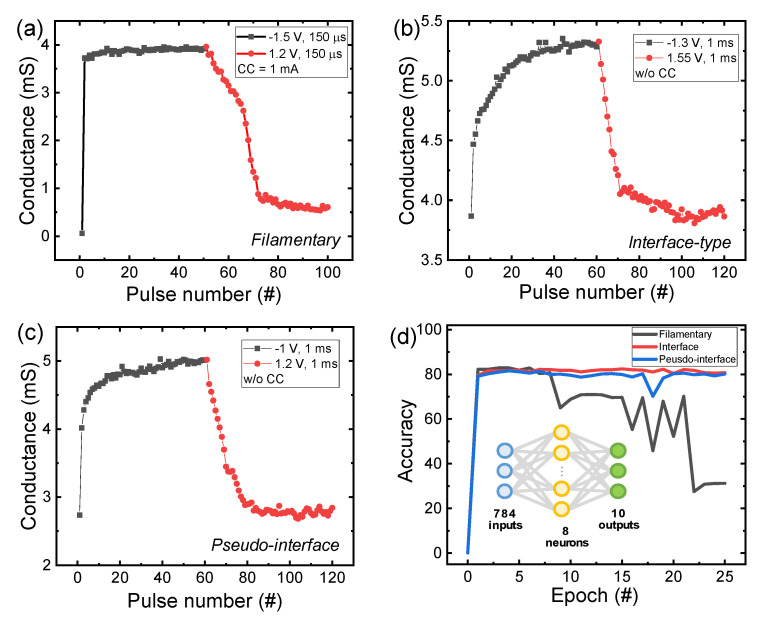
Synaptic properties of the TaO*_x_*/HfO_2_ memristor: Potentiation and depression curves: (**a**) filamentary; (**b**) interface-type; and (**c**) pseudo-interface-type switching. (**d**) Pattern recognition accuracy test for classification of images of clothing taken from the Fashion Modified National Institute of Standards and Technology (MNIST) dataset by applying the conductance of the three types of switching in the neural network.

In order to use the memristor device as a synaptic element of a neuromorphic hardware system, implementation of multi-level conductance is essential [30,31]. The three well-controlled switching models discussed above were evaluated as to their suitability for synaptic devices. Figure 5a–c show the pulse-programmed potentiation and depression curves of the three different switching cases. Negative one and a half V, 150 μs set pulses and 1.2 V, 150 μs reset pulses are applied for potentiation and depression in filamentary switching (Figure 5a). The read pulse of 0.2 V is used to extract conductance after each set or reset pulse. The tendency of the conductance value to increase rapidly after application of the first set pulse is well-matched with the abrupt set operation in DC voltage sweep. The current range is fixed at 1 mA on the equipment, and it is difficult to obtain a value for significant additional conductance values. Figure 5b shows potentiation and depression curves in interface-type switching, for which −1.3 V and 1.55 V, respectively, are applied to the devices. For interface-type switching, a longer pulse time is needed for potentiation and depression, which suggests that the interface-type switching mechanism is different from the filamentary-type switching mechanism. A gradual increase in conductance is achieved with the pulse responses. Figure 5c shows the potentiation and depression curves for pseudo-interface-type switching. Although the amount of change in the first conductance of potentiation is slightly larger, the gradually incremental conductance curve is almost close to interface-type switching are observed. In the depression curve for all three switching cases, conductance tends to converge with the pulse response repeats after the conductance changes significantly at the beginning. Figure S3 shows three repeated potentiation/depression cycles. The simplest MNIST pattern recognition is tested using a software program to evaluate the application of the three switching modes as a synaptic device [32,33]. Figure 5d shows pattern recognition accuracy with epoch for the three different switching cases. Here, the neural network is composed of 784 input layers, 8 hidden layers, and 10 output layers. We explained the neural network simulation method in more detail in a previous paper [11]. The recognition rate is greatly improved in the case of interface-type (80.79%) and pseudo-interface-type switching (80.18%) compared to when the conductance values of filament-type switching (31.25%) are entered. It is notable that pseudo-interface-type switching, with better endurance and retention properties, has almost equal pattern recognition accuracy as interface-type switching.

## 4. Conclusions

In this work, we demonstrated pseudo-interface-type switching in a Pt/TaO*_x_*/HfO_2_/TiN device in an attempt to overcome poor retention and endurance properties while maintaining good MLC characteristics. First, we characterized the current-voltage and conductance-voltage relationships of three different switching types to trace gradual and abrupt changes in switching. Next, conducting filament and defect models were presented in double insulators for pseudo interface-type switching. The oxygen vacancies which act as conducting channels inside the TaO*_x_* layer are controlled by the applied bias, so the conductance value can be changed gradually. Finally, the pattern recognition accuracy for Fashion MNIST classification was evaluated by putting the conductance values of the three switching types in a neural network. Pseudo-interface-type switching showed similar pattern recognition accuracy to interface-type switching.

## Supplementary Materials

The following are available online at https://www.mdpi.com/2079-4991/10/8/1550/s1, Figure S1: Rectifying I-V characteristics of the TaO*_x_*/HfO_2_ memristor in the LRS, Figure S2: Retention test for interface-type switching of the TaO*_x_*/HfO_2_ memristor.
