# Supplementary material for: Pseudo-Interface Switching of a Two-Terminal TaOx/HfO2 Synaptic Device for Neuromorphic Applications
Source: Nanomaterials (Basel). 2020 Aug 7;10(8):1550. doi: 10.3390/nano10081550 (PMC7466475; doi:10.3390/nano10081550)
Supplement: Supplementary file 1 [file nanomaterials-10-01550-s001.pdf]

nanomaterials

Supplementary Material

Pseudo-Interface Switching of a Two-Terminal TaO_x/HfO_2 Synaptic Device for Neuromorphic Applications

Hojeong Ryu and Sungjun Kim *

Division of Electronics and Electrical Engineering, Dongguk University, Seoul 04620, Korea; hojeong.ryu@dongguk.edu

* Correspondence: sungjun@dongguk.edu

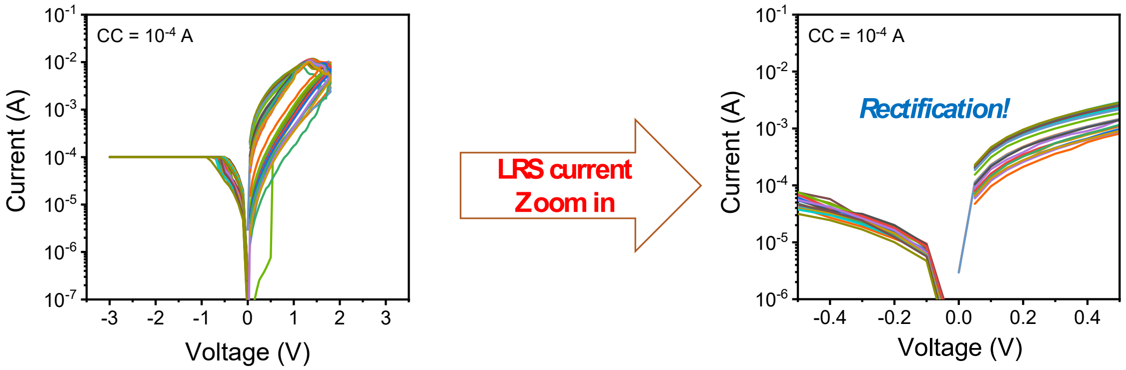

Figure S1. Rectifying I-V characteristics of the TaO_x/HfO_2 memristor in the LRS at CC of 10^{-4} A.

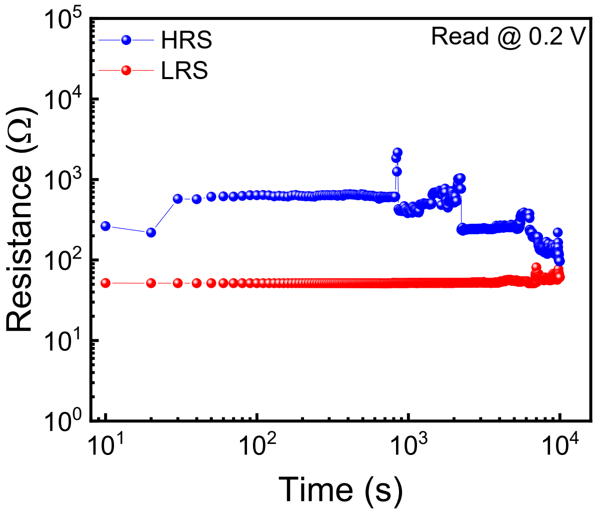

Figure S2. Retention test for interface-type switching of the TaO_x/HfO_2 memristor.

© 2020 by the authors. Licensee MDPI, Basel, Switzerland. This article is an open access article distributed under the terms and conditions of the Creative Commons Attribution (CC BY) license (http://creativecommons.org/licenses/by/4.0/).
